# The relationship between a trusted adult and adolescent outcomes: a protocol of a scoping review

**DOI:** 10.1186/s13643-018-0873-8

**Published:** 2018-11-24

**Authors:** Jan Pringle, Ross Whitehead, Dona Milne, Eileen Scott, John McAteer

**Affiliations:** 1 University of Edinburgh, 9 Hope Park Square, Edinburgh, EH8 9NW UK; 2 NHS Health Scotland, Glasgow, UK; 3 Public Health, NHS Lothian, Edinburgh, UK; 4 NHS Health Scotland, Edinburgh, UK

**Keywords:** Adolescents, Social support, Harm reduction, Educational status, Health status, Scoping review

## Abstract

**Background:**

Although documentation of harm towards children and young people has existed for centuries, it was not until the 1960s that it became a specific focus for health professionals. Since that time, the importance of protective social networks has become better understood. The concept of trusted adults has come into sharper focus, with children being encouraged to develop networks of dependable adults to turn to for support in times of need. While many child protection processes highlight risks to younger children, there has been less emphasis on older children. The role of trusted adults may be particularly important during adolescence, due to burgeoning independence, developing sexuality, relationship formation, and associated vulnerabilities. While important choices relating to health and education are made during this period, there is little formal evidence relating to the impact of trusted adults on such outcomes. This review therefore aims to focus on the role and influence of trusted adults for adolescents.

**Methods:**

This study is a scoping review. A broad range of databases will be searched, including MEDLINE, ERIC, Education Abstracts, Web of Science, ASSIA, Sociological Abstracts, and PsycINFO. Predefined inclusion/exclusion criteria will be used, with a focus on outcomes relating to health and education. Two reviewers will blind screen papers independently at all screening stages, with conflicts being resolved by a third reviewer. Quantitative and qualitative studies, as well as unpublished (grey) literature/reports, will be included. We will use the World Health Organization’s ‘second decade’ definition of adolescence. We aim to collate and map evidence in a broad overview and produce meta-analyses of homogenous data. Where this is not possible, a narrative summary will be produced.

**Discussion:**

There appears to be sparse knowledge regarding the role of trusted adults for adolescents. Potential benefits to health and wellbeing may impact on educational attainment, and vice versa. These areas are of particular relevance during the second decade, when decisions that affect future direction, achievement, and wellbeing are being made. The increased understanding of the role of trusted adults provided by this review may help to inform practice and policy and lead to potential benefits for the health and education of adolescents.

**Systematic review registration:**

PROSPERO CRD 42017076739

**Electronic supplementary material:**

The online version of this article (10.1186/s13643-018-0873-8) contains supplementary material, which is available to authorized users.

## Background

Although documentation of harm towards children and young people has existed for centuries, and received greater attention through authors such as Charles Dickens in the Victorian era, it was not the 1960s that it became a specific focus for health professionals, as marked by the publication of certain seminal work [1].

In 2002, the World Health Organization published a major overview of the impact of violence on health, highlighting the risk factors in relation to public health, and the ‘lifelong sequelae’ to health and wellbeing resulting from such harm towards children [2]. This report considered violence from a broad perspective, from individual neglect, maltreatment, and physiological abuse, through to suicide, extreme physical or sexual harm, and the violent conflict of war zones. In relation to young people, the report indicated the importance of social networks as having a protective effect and advised that children might be taught to tell an adult about any concerns or issues they might have relating to their safety.

The concept of such a trusted adult within child or adolescent health and wellbeing has come into sharper focus over more recent years. For example, the National Center for Missing and Exploited Children (NCMEC) was set up in the USA in 1984, initially to highlight the cases of missing children. The NCMEC now involves 25 participating countries worldwide, including the UK, with an expanded remit covering many aspects of child safety and abuse prevention, including highlighting the role of a trusted adult within the lives of children and young adults [3]. The need for this type of figure within the lives of children has come to be further understood as a result of child abuse scenarios, where it has become evident that such support may have prevented or limited the length or scope of harm [4].

In a further example of the role of a trusted adult, the University of the Sunshine Coast (Queensland, Australia) has worked with other child support organisations to produce a web-based resource (Orbit) designed in an informal game format to help children understand who these people might be and how they might help [5]. Within Orbit, child safety is embedded within the wider context of human rights and respectful relationships [6]; children are encouraged to develop a network of several trusted adults in order to have someone to turn to, no matter where they are. Indeed, on the Orbit website, this is stated as the ‘single most important protective factor’ against abuse [5].

Young adulthood is a particularly vulnerable time in relation to burgeoning independence, developing sexuality, and first forays into intimate relationship formation [7, 8]. While many child protection processes highlight the risks to younger children, there appears to have been less emphasis on older children [9]. Other adolescent reviews have not provided a focus on the individual support that a trusted adult might provide [10–12] or outcomes relating to health and education that having a trusted adult may impact upon for this age group [13]. Potential benefits to health and wellbeing may impact on educational attainment, and vice versa, since there are recognised connections between the two [14]. These areas are of particular relevance during the second decade, when decisions that affect future direction, achievement, and wellbeing are being made by adolescents [15, 16].

Due to the sparsity of review-level evidence, there is the potential for a detailed review to contribute to a greater understanding of the influence of having a trusted adult on these important adolescent outcomes. We therefore aim to conduct a scoping review to investigate a wide range of evidence relating to the relationship between a trusted adult and adolescent health- and education-focussed outcomes.

The broad objectives are as follows:

- To identify evidence relating to the influence of a trusted adult on health, wellbeing, and educational outcomes of adolescents
- To analyse the evidence of effects and perceptions from the above literature
- To analyse the pathways through which relationships between a trusted adult and an adolescent have an effect on health- and education-related outcomes

## Methods

We will use the Preferred Reporting Items for Systematic Reviews and Meta-Analysis Protocols (PRISMA-P) [17, 18] to chart and detail the processes and findings of the review and to ensure a structured method is employed.

### Review team

The review team is comprised of experienced researchers with a background in public health, adolescence, and systematic reviewing, plus policy/decision-makers with a remit to improve the health and wellbeing of the Scottish population.

### Inclusion and exclusion criteria

Specific study design, population, intervention/exposure, and outcome (SPIO) criteria will be applied, as detailed below.

#### Study design

The review will consider all research designs and types of publication, including quantitative (e.g. trials, observational studies) and qualitative studies as well as unpublished (grey) literature and reports, to reduce the likelihood of publication bias. Many relevant reports may have been written by charitable bodies (for example) with a remit to improve child outcomes, potentially including extensive knowledge and expertise. It was therefore considered important that such expert work was not excluded. However, inclusion will be limited to reports or studies where outcomes data are reported, rather than solely discussion or opinion pieces (unless relevant to definitions and roles, as specified above).

#### Population

Adolescents are between the ages of 10 and 19 years, based on the World Health Organization’s ‘second decade’ definition of adolescence [19], where relationships between a trusted adult (e.g. teacher, family member, support worker) are reported in relation to adolescent outcomes. If relevant findings extend beyond these years (e.g. subsequent impacts during adulthood), we will look for evidence that the trusted adult input occurred during the adolescent years. Equally, if the age range of participants incorporates and extends beyond adolescence, these studies will be included.

We will examine the findings in relation to the age range of the populations studied to ascertain if, for example, there are relationships between the timing of interventions, the age of the adolescents, and the reported outcomes.

#### Interventions

The included interventions are any study, where an intervention by (or exposure to, broader relationship with) a trusted adult has an influence or impact on an adolescent health or educational outcome. This may include studies where:

1. The relationship between a trusted adult and adolescent outcomes is qualitatively reported.
2. The relationship between a trusted adult and adolescent outcomes is quantitatively reported (e.g. cross-sectional/longitudinal associations, RCTs, other controlled trials, case studies).

For the purposes of this review, a trusted adult is defined as someone who the ‘children and young people may turn to for help and will take them seriously’ [20]. This is further defined as support from a specific, dependable (adult) individual, who acts in a responsible manner, rather than (social) support as a more general concept [5]. It is acknowledged that there may be many differing ways in which such a person or relationship is defined or termed within the literature; for example, one paper examining adverse childhood experiences defines this person as an ‘always available’ adult [21]. Other literature refers to such figures as ‘natural mentors’ [22]. As part of the review findings, we will endeavour to compile a list of terms and definitions for trusted adults, used within the included papers, to aid comparison and assist in the identification of core components of the role. For this part of the process, we may refer to non-empirical evidence that provides relevant insights into terms and definitions used in the field.

The review remit will focus on the impact that such a person, or relationship, may have on health and education outcomes of young people in their second decade.

#### Outcomes

The primary focus is on adolescent health (including general wellbeing, health behaviour, and mental health) and education-related outcomes (attainment, years in education, etc.). Outcomes later in life (e.g. in adulthood), either implicit or explicit, will also be included as secondary data, with the proviso that the trusted adult input was present during adolescence.

#### Delimiters and exclusions

To ensure currency, and to deliver within a restricted time frame, only studies from the last 10 years (i.e. 2007 onwards) will be included, as will grey literature from the past 5 years (i.e. 2012 onwards). Although an English language limitation will be applied, no geographical boundaries will be set. It is recognised that definitions of harm, support, and health/education outcomes will need to be taken in the context of individual geographical (country) location [2]. We will exclude studies where the main or sole focus involves individuals with specific, pre-existing health or learning needs (e.g. autism), due to their unique requirements.

We will also exclude studies that focus exclusively on parenting programmes, which aim to build parenting skills (generally for parents of younger children) rather than examining the role of being a trusted adult to adolescent children.

Protocols that do not report on outcomes will be excluded.

Inclusion and exclusion criteria are summarised in Table 1.

**Table 1:** Inclusion and exclusion criteria

Inclusion criteria	Exclusion criteria
• Any research design • Young adults in the 10–19-year-old age range • Interventions involving a trusted adult figure • Outcomes relating to health and wellbeing and/or education • Studies published during or after 2007	• Research protocols or reports without resulting outcomes • Children < 10 years of age, or > 19 years of age • Parenting programmes that focus solely on parenting skills • Specific pre-existing health/learning conditions (e.g. autism) • Non-human studies • Non-English language studies • Pre-2007 publications

### Data sources and search strategy

#### Electronic searches

A comprehensive search strategy that aims to be both sensitive and specific will be developed using the MEDLINE thesaurus and indexing system to identify appropriate MeSH headings and key/text words associated with trusted adults and adolescents (see Additional file 1: Appendix 1). This will be adapted for use across all included databases as necessary. It is anticipated that not all literature will refer precisely to the term ‘trusted adult’, and alternative terms of reference may need to be used, or evidence of such a role sought within individual papers/reports.

A broad range of databases will be used in the review, including MEDLINE, ERIC, Education Abstracts, Web of Science, ASSIA, Sociological Abstracts, PsycINFO, Social Services Abstracts, IBSS, the Cochrane Library, ProQuest Public Health, and SCIE Social Care Online.

#### Searching other resources

We will carry out searches in Google/Google Scholar, and contact experts in the field, to access grey literature and other relevant work not retrieved through database searches.

We will screen reference lists, and carry out citation searches of the included studies, to locate further relevant literature.

A bibliographic data management system (RefWorks™) will be used to store and organise the results of the searches.

### Study selection

Following the identification and removal of duplicates (using RefWorks™), titles and abstracts of identified papers will be screened using the broad inclusion criteria of a trusted adult and adolescent health or education outcomes. All papers that appear to fulfil these initial criteria will proceed to full-paper screening. Two of three reviewers (JP, RW, DM) will blind screen papers independently at all stages of the screening process, with any disagreements being resolved by a further review team member. This will be facilitated using Covidence.

Full-text papers/reports will be retrieved and screened using the specific study design, population, intervention, and outcome (SPIO) criteria, as noted above. Studies that do not meet the inclusion criteria will be excluded, and the reason for exclusion noted. A flow diagram will be prepared detailing and summarising the selection process and giving numbers of inclusions and exclusions at each stage.

### Data extraction and management

Data from individual studies will be extracted onto a pre-designed spreadsheet including year and location of the study, study design, study population, type of intervention/support, outcomes, and reviewer comments (e.g. relating to relevance, rigour, exclusions). This latter field will document any major design or quality flaws in the evidence. Data will be extracted independently and cross-checked by two reviewers.

### Study appraisal

We will use a mixed method appraisal tool [23] to give an indication of individual study merit. This will include an appraisal of research aims/objectives, population/sample relevance and representation, response rates, validity of findings, and bias/limitations. Study appraisal will be assessed independently by two reviewers and cross-checked to reach assessment consensus with a third reviewer, if necessary. We acknowledge that quality of reporting limitations (rather than the quality of method) may be present, particularly in grey literature. We therefore do not plan to exclude studies on the grounds of quality appraisal, other than when conducting meta-analysis, where only data that has been derived from studies that fully meet the appraisal criteria will be included. If feasible, individual authors will be contacted for further information.

We will present a tabulated summary, detailing whether appraisal criteria have been fully, partially, or not met and indicate where this has been based on published accounts only, due to lack of additional information.

### Data analysis

#### Descriptive analysis

A numerical summary and thematic analysis of the results will be conducted. The numerical summary will provide an overview of the included studies. The thematic analysis of qualitative data will include a mind-mapping approach [24] to plot, in diagrammatic form, associations and links between individual study findings. Such representation, with the trusted adult and adolescent as central figures, can help to show connections (and in this case, potential means of support) previously not apparent in purely written formats [24]. This will assist in giving an indicative synthesis of the review findings and help to develop an initial framework that may suggest the pathways through which the presence of a trusted adult can impact on adolescent outcomes. This framework would be further developed in a workshop-style consultation with key stakeholders (e.g. field experts, adolescents, teachers) to ensure relevance to practice and policy. A group of interested key stakeholders has already been identified through the liaison efforts of the review team. It is anticipated that their input will provide a basis through which initial review analysis and findings might be linked to, for example, guidance for trusted adult interventions. We would also aim to produce a user-friendly evidence map [25] in the form of a visual figure, to illustrate and summarise findings, and ensure the review findings are ‘accessible, digestible, and usable’ [26]. Both of these processes will form part of the review dissemination, alongside more formal presentations and publication for both academics and practitioners.

#### Statistical analysis

##### Measures of intervention effect

This will apply to experimental/intervention studies (e.g. RCTs). Where study outcomes are sufficiently homogenous and amenable for pooled synthesis (outcomes are measured in the same way and at similar time points), meta-analysis will be performed. We will use Review Manager 5 (RevMan5) software to assist with this process.

We will calculate risk ratios for dichotomous outcomes or standardised mean differences for continuous outcomes. For dichotomous outcomes (e.g. smoker/non-smoker; educational attainment), risk ratio (RR) and their 95% confidence interval (CI) will be calculated. For continuous outcomes (e.g. Likert wellbeing scales), mean differences (MD) with 95% CI or standardised mean differences (SMD) will be used (in the case of different measurement scales).

If there are sufficient relevant data relating to homogenous groups, or types of intervention (e.g. school-based mentoring), then cluster analysis will be considered. Cluster randomised trials will be analysed separately from individually randomised trials to prevent an underestimation of the standard error of the effect estimate, due to a unit of analysis issues. We will use the design effect method described by Eldridge and Kerry [27]. Some cluster trials may not be adjusted for clustering in their analysis, so we may attempt to estimate the intra-cluster correlation coefficient (ICC).

If the authors do not provide the necessary data in the paper, we will write to them to request this data. If we are unable to obtain the data, we will use the adjusted results and be guided by the processes combining adjusted and unadjusted findings in mixed research synthesis [28].

##### Heterogeneity

We will assess heterogeneity by visual inspection of forest plots and by the use of the statistical *I*^2^ test for heterogeneity *I*^2^ [29]. *I*^2^ values ranging from 0% (homogeneity) to 100% (heterogeneity) will be calculated to quantify variability in study effect. An *I*^2^ value of greater than 50% will be considered the cut point for significant heterogeneity [29]. Where possible, subgroup analyses will be performed to explore and explain the heterogeneity among studies [30].

Subgroup analyses, where appropriate, will be based on health and wellbeing outcomes (including mental health and health behaviours) and educational outcomes (e.g. years in education, educational attainment, future prospects such as university attendance and work placements).

#### Cumulative evidence

We will aim to provide a descriptive summary of the cumulative evidence from this review, including any gaps in evidence. Following completion of evidence mapping and establishing if meta-analysis is feasible, we will be able to further define the specific questions the review is able to address.

## Discussion

This review seeks to provide a comprehensive overview of the role of a trusted adult on health and education outcomes for adolescents. It will help to collate evidence relating to the value that such a role can bring to this life stage. While promoting the wellbeing and health of children has been recognised as a priority for educational attainment at school [31], previous reviews have not given sufficient attention to the impact and value that the role of a trusted adult may have for adolescents [10–12]. This is particularly important, given the decisions that are made and foundations that are laid down during this period [15, 16]. The review will build on the work of other reviews that examine school programmes in a more general way across all school age ranges [14] and the constraints that may inhibit such trusted relationships from developing [32].

It is felt that evidence provided by this review will be vital to inform and encourage support interventions by trusted adults for adolescents, including details of the nature and scope of the relationship that may have been previously lacking.

### Relevance of the review

While the need to support adolescents during their transition to the independent roles of adulthood has been well-documented [16, 30], additional information relating to the specific nature of such support is of practical use to those in the role of providing or influencing delivery. Such support has the potential to lead to the reduction of harm, and the improved future wellbeing and potential of adolescents [5]. Improving the health and attainment of adolescents has positive implications for both their future and society as a whole [33].

### Strengths and limitations of the review

The review will use a clear, robust, and systematic approach to searching, screening, and reviewing studies. However, the search strategies and engines may still not capture all relevant material. The additional search methods that will be employed (e.g. reference list screening and citation searches) will act to ameliorate such limitations.

The findings may be open to selection bias. However, the presence of the wider project research team, who will assist in the event of inclusion uncertainty, will serve to minimise any partiality.

The inclusion of, and input from, the wider stakeholder group will contribute to developing conclusions from the findings that have relevance in a broader practice and policy context, thereby promoting the worth of the review.

## Additional file

Additional file 1: Appendix 1. Search strategy. (DOCX 19 kb)
