# Supplementary material for: The relationship between a trusted adult and adolescent outcomes: a protocol of a scoping review
Source: Syst Rev. 2018 Nov 24;7:207. doi: 10.1186/s13643-018-0873-8 (PMC6260676; doi:10.1186/s13643-018-0873-8)
Supplement: Supplementary file 1 — Appendix 1. Search strategy. (DOCX 19 kb) [file 13643_2018_873_MOESM1_ESM.docx]

Search strategy

**Pillars:**

Population pillar: adolescent related terms

Intervention pillar: ‘trusted’ adult input/role

Outcome pillar: Health, well-being and/or educational outcomes

Devised using MEDLINE thesaurus and MeSH terms and definitions

1. adolescent/
2. adolescen$.tw
3. teen$.tw
4. youth$.tw
5. or/1-4
6. social support/
7. preventive health services/
8. trust/
9. interpersonal relations/
10. social worker/
11. mentors/
12. counseling/
13. social support$.tw
14. adult support.tw
15. support$.tw
16. preventive health service$.tw
17. trust$.tw
18. trusted adult.tw
19. social work$.tw
20. youthwork$.tw
21. mentor$.tw
22. interperson$.tw
23. coach$,tw
24. counsel$.tw
25. named person$.tw
26. youth-adult-relationship$.tw
27. or/6-26
28. adolescent health/
29. adolescent development/
30. health status/
31. mental health/
32. child welfare/
33. child abuse/
34. safety/
35. harm reduction/
36. health promotion/
37. education/
38. educational status/
39. learning/
40. social capital/
41. achievement/
42. resilience, psychological/
43. adolescen$ health.tw
44. adolescen$ development$.tw
45. health status.tw
46. mental health.tw
47. child welfare.tw
48. child abus$.tw
49. safety.tw
50. harm reduc$.tw
51. health promot$.tw
52. educat$.tw
53. educational status.tw
54. learn$.tw
55. social capital.tw
56. achieve$.tw
57. resilience.tw
58. attain$.tw
59. well-being.tw
60. or/28-59
61. 5 AND 27 AND 60 NOT autistic disorder/ autism.tw or learning disorders/ learning support.tw

**Limiters**: date of publication 2007 onwards (grey literature from 2012); English language; human not animal
